# Biosorption of Reactive Dyes by Novel Bacterium *Leclercia adecarboxylata* : Complete Removal of Reactive Black 5 and Molecular Insights Into the Adsorption Mechanism

**DOI:** 10.1002/wer.70109

**Published:** 2025-06-09

**Authors:** Seda Şen, Filiz Korkmaz, Nur Koçberber Kiliç

**Affiliations:** ^1^ Department of Biology, Faculty of Science Ankara University Ankara Turkey; ^2^ Ankara University of Natural and Applied Science Ankara Turkey; ^3^ Biophysics Laboratory, Faculty of Engineering Atilim University Ankara Turkey

**Keywords:** biosorption, isotherm, kinetic models, *Leclercia adecarboxylata*, reactive black 5

## Abstract

*Leclercia adecarboxylata* isolated from the Düden Waterfall (Turkey) was utilized as a biosorbent for the removal of Reactive Black 5 (RB5), Setazol Blue BRF‐X (BRF‐X), Setazol Navy Blue SBG (SNB), and Setazol Turquoise Blue G (STBG). Of the dyes, RB5 was removed with the highest efficiency, 97.4% after 60 min. The effect of parameters such as pH (3–9), initial biosorbent dose (0.1–2.0 g/L), and initial dye concentration (25–1200 mg/L) on the biosorption of RB5 was investigated. Increasing the biosorbent dosage from 0.1 to 2.0 g/L enhanced the RB5 removal from 55.3% to 100% within 10 min. The complete removal (100%) of RB5 was achieved in media with 2.0 g/L biosorbent and 25 mg/L RB5 at pH 3 after 10 min. Additionally, the soluble extracellular polymeric substances (EPS) of *L. adecarboxylata* were found to consist of proteins, lipids, nucleic acids, and polysaccharides according to Fourier transform infrared spectroscopy (FTIR) analysis. The EPS was found to play a crucial role in dye removal, forming chemical interactions with dye molecules. Zeta potential analysis was used to evaluate the charge distribution on the biosorbent surface (−12.6 ± 1.1 mV) and its interactions in the biosorption process. Kinetic and isotherm models suggested a complex interaction mechanism between the biomass and the dye. Adsorption isotherm data were analyzed via nine isotherm models. Among them, the Hill model was found to be the best fit for describing the equilibrium adsorption process of the RB5 (*R* ^2^ = 0.9993). Overall, the applied models elucidated the influence of both physical and chemical interactions on the mechanism. Kinetic studies revealed that the adsorption of RB5 fit a pseudo‐second‐order kinetic model. The unique biochemical composition of the indigenous *L. adecarboxylata* biosorbent provided a high affinity for RB5, offering a sustainable, rapid, and economical solution for the treatment of dye‐polluted water.

## Graphical abstract

Reactive Black 5 was biosorbed by *Leclercia adecarboxylata* , highlighting removal and adsorption mechanism.

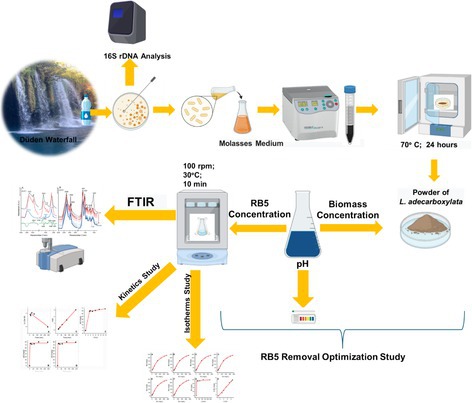

**Summary**

-
*Leclercia adecarboxylata* isolated from the Düden Waterfall (Türkiye) was used as a biosorbent to remove Reactive Black 5 (RB5) dye.
- A 100% removal of RB5 was achieved in media with 2.0 g/L biosorbent concentration at pH 3, 25 mg/L RB5 concentration, for a contact time of 10 min.
- The Hill model was found to be the best fit for describing the equilibrium adsorption process of RB5 dye (*R* ^2^ = 0.9993).
- Kinetic studies showed that the adsorption of RB5 fitted the pseudo‐second‐order kinetic model.

**Abbreviations**
*a*_*R*_
: Redlich–Peterson isotherm constant (1/mg)
AVB: *Aspergillus versicolor* biomass
*a*_*S*_
: Sips isotherm constant (L/mg)
*a*_*T*_
: Toth isotherm constant (L/mg)
*b*
: Langmuir isotherm constant or affinity constant (dm^3^/mg)
BRF‐X: Setazol blue BRF‐X dye
*b*_*T*_
: Temkin isotherm constant, which is related to the sorption heat (J/mol)
*C*
: concentration (mg/L)
*C*_*e*_
: equilibrium concentration of adsorbate on the adsorbent (mg/L)
CTAB: cetyl trimethyl ammonium bromide
DR23: Direct Red 23 dye
EBT: Eriochrome Black T dye
EPS: extracellular polymeric substances
IPD: intraparticle diffusion models
*K*_*F*_
: Freundlich constant
*K*_*H*_
: Hill isotherm constant
*K*_*Ha*_
: Halsey constant
*K*_*L*_
: Langmuir adsorption constant (L/mg)
*K*_*R*_
: Redlich–Peterson isotherm constant (L/g)
*K*_*S*_
: Sips isotherm model constant (L/g)
MG: malachite green
nano‐ZnO/CT‐CB: nano‐ZnO/chitosan composite beads
*n*_*H*_
: Hill cooperativity coefficient of the binding interaction
*n*_*R*_
: Redlich–Peterson exponent that lies between 0 and 1
*q*
: adsorption capacity (mg/g)
*q*_*e*_
: amount of the adsorbate at equilibrium (mg/g)
*q*_*m*_
: maximum uptake saturation
RB5: reactive Black 5 dye
RR120: reactive Red 120 dye
RSS: residual sum of squares
SB15: sulfur blue 15 dye
SNB: Setazol Navy Blue SBG dye
SO₃^−^
: sulfonic groups
STBG: Setazol Turquoise Blue G dye

## 1 Introduction

Water is crucial for the survival of all organisms. In recent years, human activities such as agriculture, urbanization, technology, and industrialization have significantly contaminated water sources. Industries, including textile dyes, detergents, additives, aldehydes, heavy metals, and biologically persistent organic and inorganic materials, are major contributors to ecosystem pollution (Ahlawat et al. 2020; Chong and Tam 2020). Dyes are considered as micropollutants in aquatic environments at concentrations also below 1 mg/L (Sharma et al. 2021). These dyes encompass various types, such as basic, reactive, anthraquinone, disperse, acidic, azo, diazo, and metal‐complex (Mollaie et al. 2025; el Benkhaya et al. 2017). Furthermore, the presence of these resistant dye molecules can cause long‐term adverse effects on aquatic ecosystems, such as increased toxicity, reduced dissolved oxygen levels, turbidity, and accumulation in the food chain (Mengelizadeh et al. 2025).

When consumed or inhaled, textile dyes can cause acute toxicity in humans, leading to skin and eye irritation (Reddy and Osborne 2020). Even at low concentrations, dyes in water can have carcinogenic effects on organisms (Ihsanullah et al. 2020). As a result, the treatment of wastewater containing such pollutants is imperative. Various physicochemical methods, such as ion exchange, oxidation, ozonation, coagulation, membrane filtration, solid‐phase extraction, photocatalysis, and electrochemical degradation, have been developed for pollutant removal (Dolatabadi et al. 2020; Abdollahi et al. 2012; Awwad et al. 2020). However, traditional methods suffer from drawbacks, including low efficiency, high costs, complex operational procedures, significant secondary sludge production, and limited scalability for commercial applications (Ihsanullah et al. 2020). To ensure the long‐term health of the environment, it is crucial to adopt low‐cost, eco‐friendly, and sustainable methods for removing these pollutants from water. Biological treatment methods for dye removal offer advantages over physical and chemical techniques in terms of efficiency, cost‐effectiveness, and sustainability (Biswas and Basak 2021). The basis of biological treatment lies bioremediation, which leverages the natural biological mechanisms of microorganisms to degrade various pollutants. Microorganisms capable of bioremediation can detoxify or metabolize toxic substances, converting them into nontoxic compounds (Mustafa et al. 2021). Therefore, bioremediation has emerged as a pivotal approach for mitigating and preventing environmental pollution. Among bioremediation pathways, biosorption stands out as a rapid, eco‐friendly, and cost‐effective process where molecules such as metal ions and dyes are passively removed from the environment by dead or inactive microbial cells (Ravindiran et al. 2019). Numerous bacterial, fungal, yeast, and algal species have been investigated as biosorbents for pollutant removal owing to their rapid isolation and suitability for biological processes, particularly in the removal of organic pollutants (Bilal and Iqbal 2020).

For example, Huang et al. (2016) investigated the use of cetyl trimethyl ammonium bromide (CTAB)‐modified *Aspergillus versicolor* biomass (AVB) for the removal of Reactive Black 5 (RB5) and examined parameters such as the CTAB concentration, pH, and contact time to determine the most effective removal rate. They achieved an RB5 removal above 98% with 1.5% CTAB‐modified AVB at pH 2 after 420 min of contact time for an initial dye concentration of 200 mg/L. Kamalian et al. (2024) investigated the effects of varying pH, nanoparticle dose, dye concentration, and stirring speed on removing RB5 and Direct Red 23 dyes from aqueous solutions via biogenic copper oxide nanoparticles synthesized from *Stenotrophomonas* sp. They reported a 94% removal efficiency for an initial dye concentration of 20 mg/L and a nanoparticle dose of 0.08 g after 80 min while stirring the mixture at 500 rpm. In the study conducted by Çınar et al. (2017), the removal of RB5 from aqueous solutions using nano‐ZnO/chitosan composite beads (nano‐ZnO/CT‐CB) was investigated, with a focus on experimental parameters such as contact time, pH, and temperature. That study revealed that under optimal conditions (pH 4 and adsorbent concentration of 0.2 g), dye removal was 76%.

Biosorption studies of other toxic dyes by microbial sorbents have also been reported in the literature. For example, Nguyen et al. (2016) investigated the removal of Sulfur Blue 15 (SB15) dye by the *Acidithiobacillus thiooxidans* biosorbent. They reported that 50% biosorption of SB15 was found at 300 mg/L dye concentration and 1.0 g/L biomass concentration at pH 11.7. Sarim et al. (2019) studied the biosorption of Congo Red with using *Bacillus subtilis* KK01 isolated from contaminated soil, reporting optimal conditions such as pH 7, 35°C, and 1% inoculum for the highest biosorption of 92.8%. Reddy and Osborne (2020) utilized calcium alginate beads immobilized with *Pseudomonas guariconensis* VITSAJ5 for the removal of Reactive Red 120. They reported that compared with free bacterial cells, immobilized bacterial cells removed 87% of RR120 from the media whereas free bacterial cells removed a maximum of 37%.

The aim of this study was to investigate the biosorption of four different reactive dyes, namely, Reactive Black 5 (RB5), Setazol Blue BRF‐X (BRF‐X), Setazol Navy Blue SBG (SNB), and Setazol Turquoise Blue G (STBG), from aqueous solutions using a new bacterium, *Leclercia adecarboxylata* , as a biosorbent. Among the bacteria isolated from Düden Waterfall (Türkiye), it was selected for biosorption experiments. The reason for this was that *L. adecarboxylata* produced high biomass in a shorter time compared to other isolated bacteria. High biomass production is an important factor in the biosorption process. Of the tested dyes (Reactive Black 5 [RB5], Setazol Blue BRF‐X [BRF‐X], Setazol Navy Blue SBG [SNB], and Setazol Turquoise Blue G [STBG]), RB5 was selected in the current investigation. This is because *L. adecarboxylata* biosorbent removed the RB5 from the media in the shortest contact time and with maximum biosorption capacity compared to other dyes. Dye biosorption by the dry biomass of *L. adecarboxylata* was investigated with respect to different pH values, dye concentrations, and initial biomass concentrations. Another purpose of our study was to understand the dye removal mechanism at the molecular level via FTIR spectroscopy and to employ isotherm models and kinetic studies to improve our understanding of the biosorption mechanism.

To the best of our knowledge, this study uniquely reports the use of *L. adecarboxylata* , a novel and local strain isolated from Düden Waterfall (Türkiye), as a biosorbent for the removal of reactive dyes (RB5, BRF‐X, SNB, and STGB). This study, conducted with the *L. adecarboxylata* , is innovative in that it identifies this strain as the most effective biosorbent for RB5 reported to date. It not only demonstrates its high biosorption performance but also provides a comprehensive molecular‐level analysis of the adsorption mechanism.

## 2 Materials and Methods

### 2.1 Isolation of Bacteria and Growth Conditions

Water samples from the Düden Waterfall (Antalya, Turkey) were enriched through periodic subculturing in nutrient broth (NB, Neogen, United States) supplemented with 50 mg/L RB5 dye. A 0.1‐mL portion of the enriched culture was plated on nutrient agar (1.5% w/v) and incubated at 30°C. Various colony morphotypes were observed and isolated. Pure bacterial cultures were subsequently transferred to agar slants and stored at 4°C, and the media was changed every 3 months. Among these strains, strain X3 was selected and identified based on its high growth.

### 2.2 Biosorbent Preparation From *L. adecarboxylata*

*L. adecarboxylata* was cultivated in media supplemented with 8% molasses at 30°C in a rotary shaker (NB 205 V, Korea) operating at 100 rpm. The bacterial cells were separated from the culture medium via centrifugation at 6000 rpm for 10 min via a Hermle Z207A centrifuge (Germany). The collected bacterial pellet was subsequently dried in an oven at 70°C for 24 h in glass Petri dishes. The dried bacteria were then harvested by scraping from Petri dishes and used in biosorption studies.

### 2.3 Effects of Different Parameters on Dye Biosorption

**FIGURE 1:**
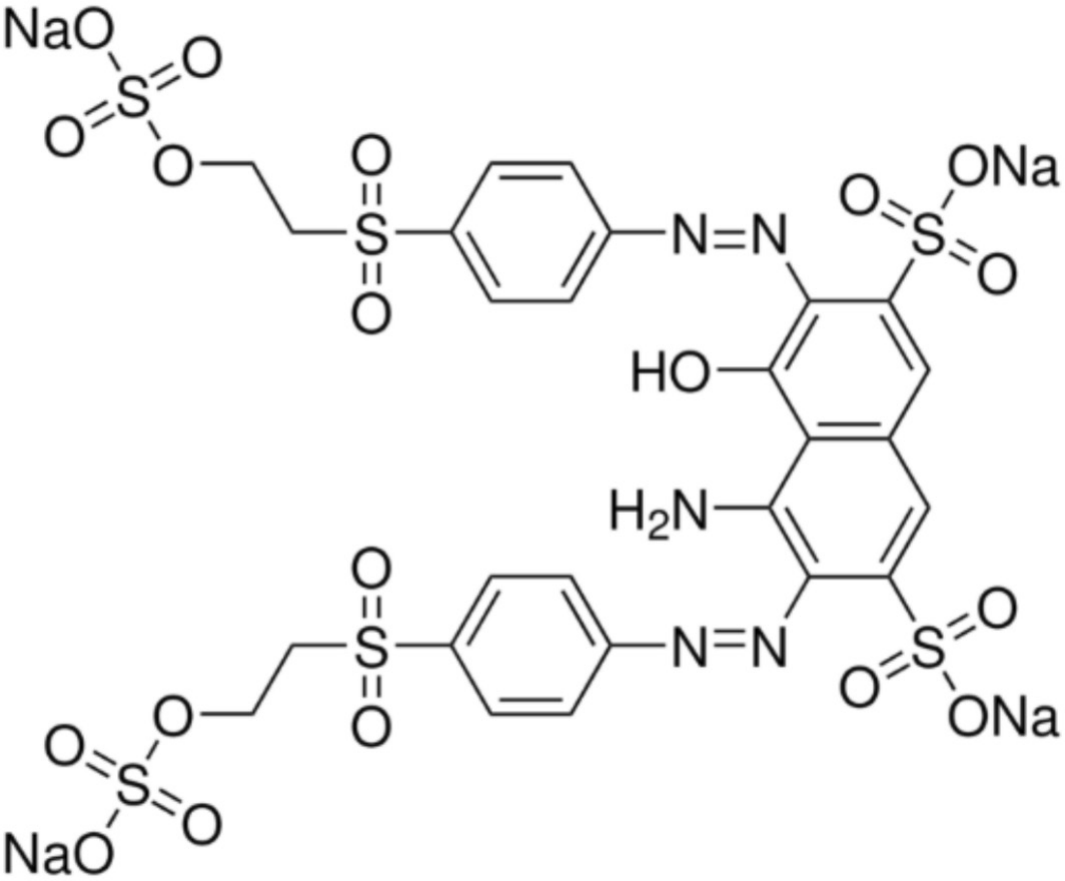
Molecular structure of RB5.

All the experiments were carried out in 40‐mL solutions prepared in 100‐mL conical flasks at six different time intervals (10, 15, 30, 60, 120, and 240 min) at a shaking speed of 150 rpm at 30°C. After incubation, the residual dye concentration in the medium was measured spectrophotometrically (BioDrop, United Kingdom). The molecular structure of RB5 was shown in Figure 1. The pure dyes used were purchased from Setaş Kimya (Tekirdağ, Turkey).

In the experiments, media containing 25 mg/L of Reactive Black 5 (RB5), Setazol Blue BRF‐X (BRF‐X), Setazol Navy Blue SBG (SNB), or Setazol Turquoise Blue G (STBG) dyes were prepared. The trials were performed at pH 3. When removal was complete, the remaining dye concentrations in the media were determined spectrophotometrically by following their signature peaks at 595 nm for RB5, at 609 nm for BRF‐X, at 669 nm for STBG, and at 602 nm for SNB. The dye that was most efficiently removed from the media by the biosorbent was identified.

To determine the optimal pH for maximal biosorption, a range of values between 3 and 9 was tested. Solutions with an initial dye concentration of 25 mg/L were prepared at seven different pH levels, that is, pH 3.0, pH 4.0, pH 5.0, pH 6.0, pH 7.0, pH 8.0, and pH 9.0.

Five separate media were prepared with a constant 25 mg/L RB5 concentration at the optimum pH. Next, different concentrations of biosorbent (0.1, 0.25, 0.5, 1.0, and 2.0 g/L) were added to each flask to determine their effects on RB5 removal.

Nine different media were prepared with a constant concentration of 2.0 g/L biosorbent at the optimum pH. A variety of dye concentrations were used to examine their effects on biosorption efficiency. These concentrations were 25, 50, 100, 150, 300, 600, 800, 1000, and 1200 mg/L. To investigate the effects of increasing dye concentrations on the biosorption efficiency, solutions containing RB5 at nine different concentrations (25, 50, 100, 150, 300, 600, 800, 1000, and 1200 mg/L) were prepared.

### Analytical Methods

During the incubation period, a 3‐mL sample was taken. The aliquots of these samples were centrifuged at 6000 rpm for 10 min to harvest the biomass. The remaining concentration of the pollutant in the medium was determined spectrophotometrically. Pollutant removal was studied as a function of pH, initial biomass concentration, and initial dye concentration, and the removal percentage was calculated via Equation (1):

(1) $$\text{Removal}\;\%=\frac{\left(C_{0}-C_{f}\right)}{C_{0}}\times 100.$$

Pollutant removal can be measured based on the mass balance principle with Equation (2) (Aksu et al. 2007):

(2) $$q_{m}=\left[\left(C_{0}-C_{f}\right)\right]÷X_{m}.$$

The maximal specific pollutant removal (*q*_*m*_) represents the maximal amount of pollutant (mg) per unit of dry weight of the bacterial cells (g). The maximum dry bacterial cell mass was *X*_*m*_ (g/L), *C*_0_ was the initial concentration, and *C*_*f*_ was the final dye concentration (mg/L).

### 2.4 Statistical Analysis

All experiments were conducted using a completely randomized design with two replicates. The data were analyzed to determine significant differences among treatment means and presented as mean values with standard errors (± SE).

### 2.5 PCR and Sequencing

The identification of bacterial cells involved amplification of the 16S rDNA gene. Three microliters of cells was added directly to polymerase chain reaction (PCR) tubes using the bacterial primers 341F and 805R to amplify the V3 and V4 regions of the 16S rDNA gene (Hugerth et al. 2014). An initial denaturation step at 95°C for 3 min was followed by 35 cycles of denaturation at 95°C for 20 s, annealing at 57°C for 25 s, and extension at 72°C for 50 s in the PCR. Finally, a 5‐min elongation at 72°C was used to terminate the cycle. Sequencing of the samples (Applied Biosystems, Foster City, CA) was performed via an ABI 3130XL Sanger sequencing instrument and the ADS SupreDye v3.1 Cycle Sequencing Kit. BLAST was used to perform the phylogenetic analysis of the nearly complete data. The neighbor‐joining method in MEGA 11.0 software was also used to carry out further analysis (Kumar et al. 2018).

### 2.6 Scanning Electron Microscopy (SEM)

The morphology of *L. adecarboxylata* dry biomass was analyzed by scanning electron microscopy (SEM). Prior to imaging, the biomass samples were coated with a 17‐nm carbon layer to ensure conductivity (GAIA3 SEM equipped with an Oxford XMax 150 EDS instrument).

### 2.7 Zeta Potential Measurements

The zeta potential of *L. adecarboxylata* dry biomass was measured to evaluate its surface charge and electrostatic properties. Zeta potential measurements were performed via a Malvern Zetasizer Nano ZSP device.

### 2.8 Adsorption of Kinetics and Isotherms

Biosorption kinetics were studied via the pseudo‐first‐order, pseudo‐second‐order, and intraparticle diffusion (IPD) models, expressed by the equations tabulated in Table 1. Ten different isotherm models were tested to understand the adsorption mechanism and the properties of the adsorbent and the adsorbate. The nonlinear forms of the isotherm models were used except for the Halsey model. Fits were performed, and visual images were prepared in OriginPro 2017 (Massachusetts, United States).

**TABLE 1:** Adsorption kinetic and isotherm models.

	Nonlinear form	Linear form	Constants
Pseudo 1st order	$q_{t}=q_{e}\left(1-e^{-k_{1}t}\right)$	$\ln\left(q_{e}-q_{t}\right)=\ln\left(q_{e}\right)-k_{1}t$	$k_{1}$ (min^−1^)
Pseudo 2nd order	$q_{t}=\frac{q_{e}^{2}k_{2}t}{1+q_{e}k_{2}t}$	$\frac{t}{q_{t}}=\frac{1}{k_{2}q_{e}^{2}}+\frac{t}{q_{e}}$	$k_{2}$ (g/mg min)
Intraparticle diffusion (IPD)	$q_{t}=k_{\mathrm{IPD}}\sqrt{t}+C$		$k_{\mathrm{IPD}}$ (mg/g min^1/2^) C (mg/g)
Elovich isotherm	$q_{t}=\frac{1}{\beta }\ln\left(1+\mathrm{\alpha \beta t}\right)$		α (mg/g min) β (g/mg)
Freundlich isotherm	$q_{e}=K_{F}{C_{e}}^{1/n}$		$K_{F}$ (unitless)
Hill isotherm	$q_{e}=q_{m}\frac{{C_{e}}^{n_{H}}}{K_{H}+{C_{e}}^{n_{H}}}$		$K_{H}$ (unitless) $q_{m}$ (mg/L)
Halsey isotherm	$q_{e}=e^{\left(\ln K_{\mathrm{Ha}}-\ln C_{e}\right)/n_{\mathrm{Ha}}}$	$\ln q_{e}=\frac{1}{n_{\mathrm{Ha}}}\ln K_{\mathrm{Ha}}-\frac{1}{n_{\mathrm{Ha}}}\ln C_{e}$	$K_{Ha},n_{Ha}$ (unitless)
Jovanovich isotherm	$q_{e}=q_{m}\left(1-e^{-K_{J}C_{e}}\right)$		$K_{J}$ (L/mg) $q_{m}$ (mg/g)
Langmuir isotherm	$q_{e}=\frac{q_{m}K_{L}C_{e}}{1+K_{L}C_{e}}$		$K_{L}$ (L/mg) $q_{m}$ (mg/g)
Redlich–Peterson isotherm	$q_{e}=\frac{K_{R}C_{e}}{1+a{C_{e}}^{n_{R}}}$		$K_{R}$ (L/g) a (L/mg)
Sips isotherm	$q_{e}=\frac{q_{m}{\left(K_{S}C_{e}\right)}^{n}}{1+{\left(K_{S}C_{e}\right)}^{n}}$		$K_{S}$ (L/g) $q_{m}$ (mg/g)
Temkin isotherm	$q_{e}=\frac{\mathrm{RT}}{b_{T}}\ln\left(A_{T}C_{e}\right)$		$A_{T}$(L/mg) $b_{T}$ (J/mol) T = 302 K R = 8.314 J/molK
Toth isotherm	$q_{e}=\frac{q_{m}K_{T}C_{e}}{{\left(1+{K_{T}C_{e}}^{t}\right)}^{1/t}}$		$K_{T}$ (unitless) $q_{m}$ (mg/g)

### 2.9 Fourier Transform Infrared Spectroscopy (FTIR) Analysis

ATR‐FTIR spectroscopy was used to characterize the biomass with or without RB5. The biomass + dye sample was centrifuged to separate the biomass from its environment. Both the pellet and the supernatant were analyzed to observe the functional groups that interact with the dye molecules. A Thermo‐Scientific Nicolet 6700 FTIR spectrometer equipped with a diamond, 10‐bounce attenuated total reflection (ATR, ConcentratIR2, Harrick) accessory was used to obtain the infrared spectra. Fifty scans were averaged for 4‐cm^−1^ resolution. The spectra were ATR corrected via a built‐in function in the spectrometer software OMNIC (v. 8.2.388).

## 3 Results and Discussion

### 3.1 Identification of Bacteria

**FIGURE 2:**
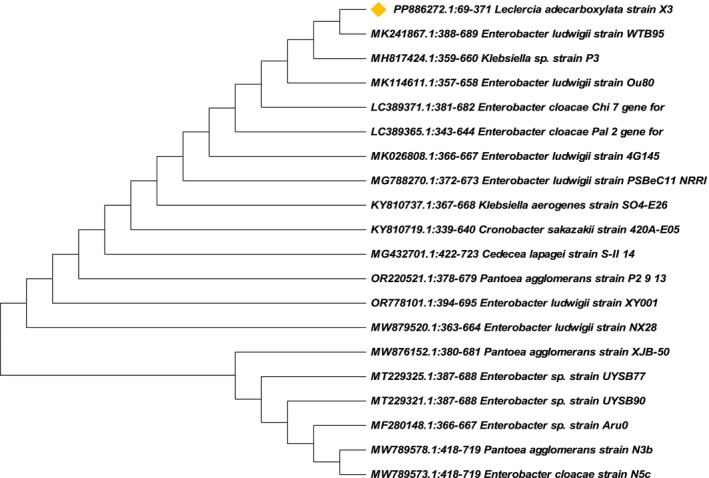
Phylogenetic tree of *L. adecarboxylata* X3.

The isolate (X3) was identified by amplification and sequencing of its 16S rDNA gene. A BLAST search was performed for phylogenetic analysis of the nearly complete sequence data. Alignment and further analysis revealed 100% similarity to *L. adecarboxylata* . The evolutionary history was inferred via the neighbor‐joining method (Saitou and Nei 1987), and the optimal tree is shown in Figure 2. The percentage of replicate trees in which the associated taxa clustered together in the bootstrap test (100 replicates) is shown next to the branches (Felsenstein 1985). The tree was drawn to scale, with branch lengths in the same units as those of the evolutionary distances used to infer the phylogenetic tree. The evolutionary distances were computed via the maximum composite likelihood method (Tamura et al. 2004) and were expressed in units of the number of base substitutions per site. This analysis involved 20 nucleotide sequences. All ambiguous positions were removed for each sequence pair (pairwise deletion option). There was a total of 304 positions in the final dataset. Evolutionary analyses were conducted in MEGA (Tempe, AZ, United States) (Tamura et al. 2021). The bacterium was submitted to NCBI GenBank with the accession number *L. adecarboxylata* PP886272.

### 3.2 Morphological Characterization (SEM)

**FIGURE 3:**
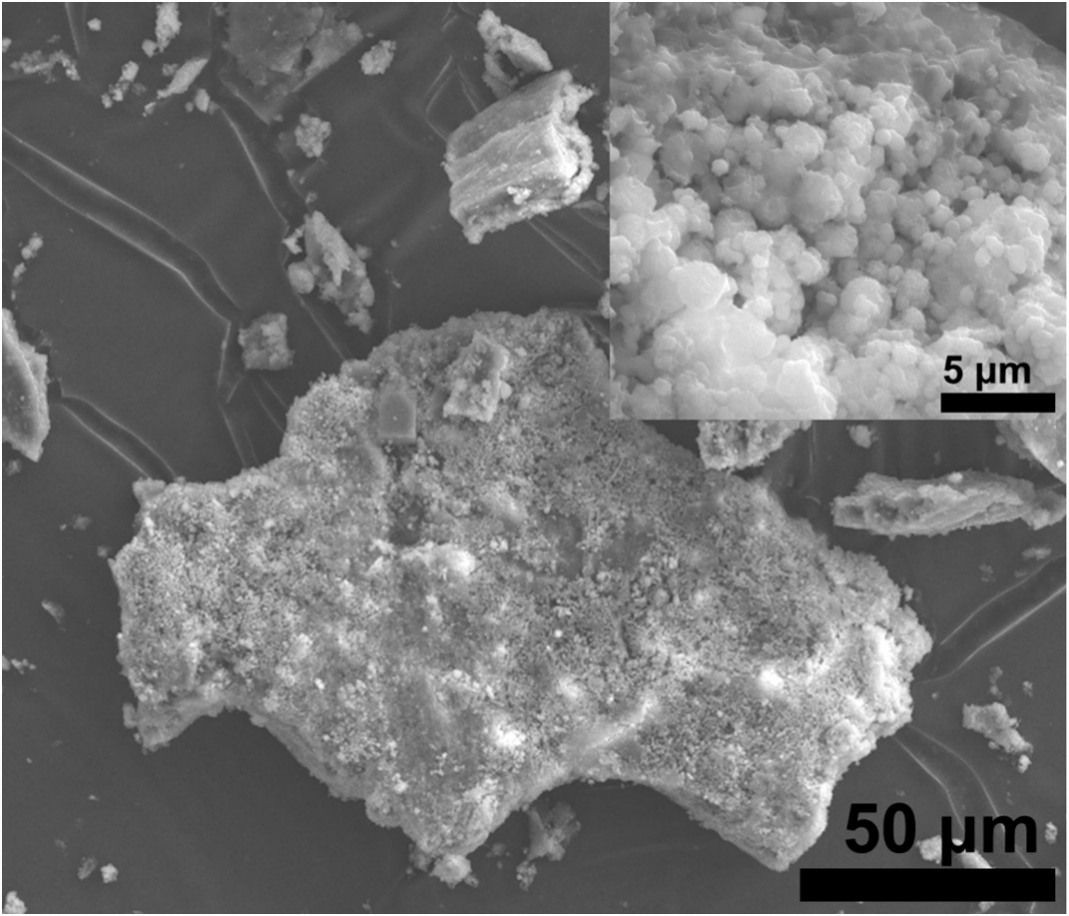
Scanning electron micrograph of the *L. adecarboxylata* dry biomass.

The morphological characteristics of *L. adecarboxylata* dry biomass were analyzed by SEM. The obtained SEM images were presented in Figure 3.

The biosorbent particles were composed of irregular crystalline forms that exhibit a network‐like structure on their surface. The dye molecules in the medium adsorbed to this structure were identified in the SEM micrograph. Similarly, in a previous study, a comparable structure consisting of irregular crystalline forms suitable for pigment adsorption was observed and confirmed on the surface of an *Enterobacter* sp. biosorbent (Muthulakshmi et al. 2021).

### 3.3 Biosorption of Different Dyes

**FIGURE 4:**
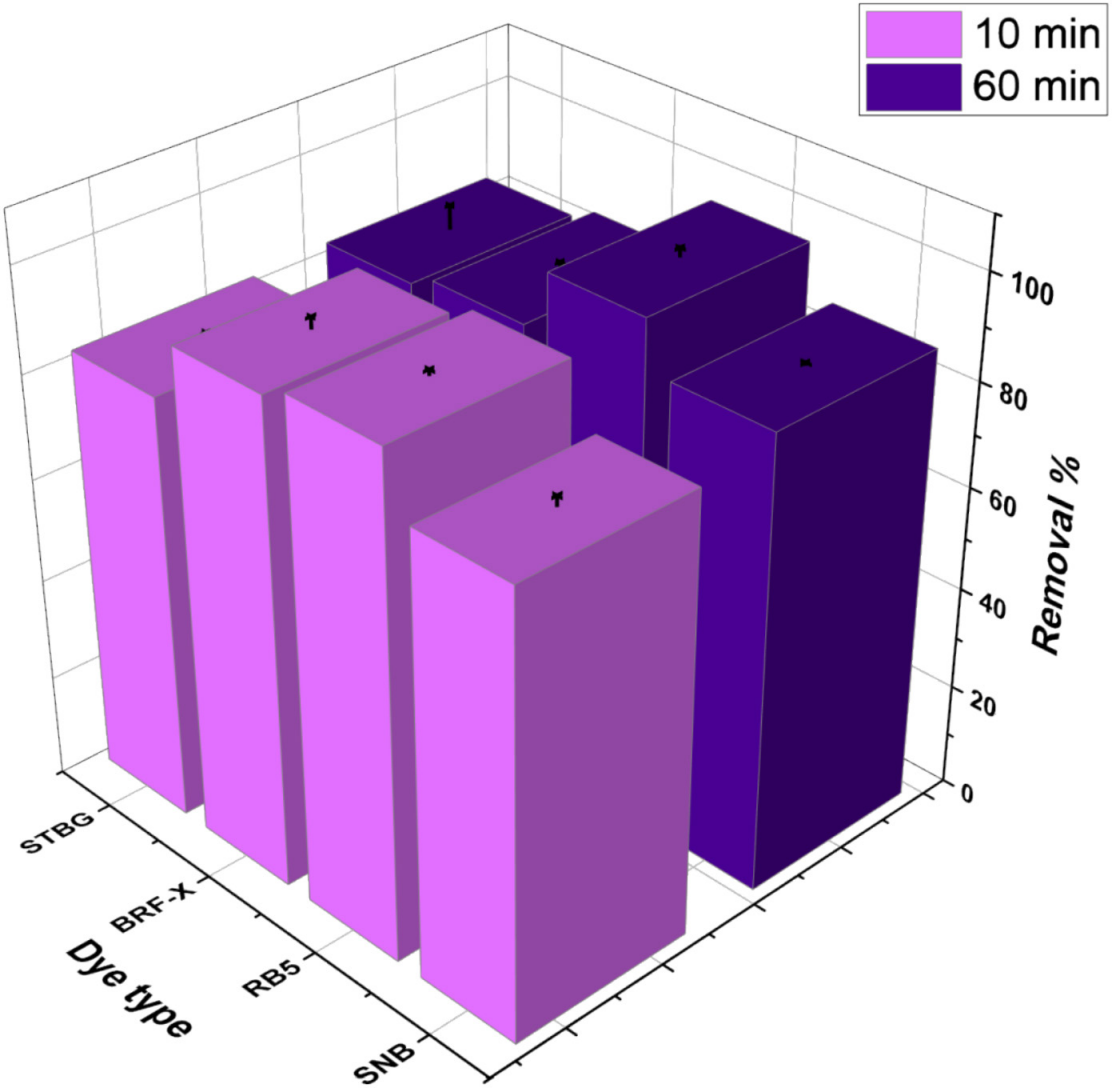
Biosorption of four different dyes by *L. adecarboxylata* biomass in aqueous solution recorded at 10 and 60 min (pH: 3; *C*_0_: 25 mg/L; *T*: 30°C; volume: 40 mL; biomass dose: 0.25 g/L).

The experiments were carried out to identify the dye that *L. adecarboxylata* can remove most effectively. Four different dyes were tested, and their removal rates were recorded at two different time points during contact time. All the data obtained in this series were shown in Figure 4.

The removal percentages for all the tested dyes reached above 80% after 10 min. The highest removal rates were recorded for the RB5 dye, which was removed by 96.5% after 10 min and by 97.4% after 60 min. Only a slight improvement in the removal rate after prolonged contact time argues for the electrostatic nature of the interaction between the dye molecules and the biomass. Each dye used in this study was anionic and negatively charged in aqueous solutions, whereas the net charge of the tested biosorbent was positive at pH 3. The higher removal rate recorded for RB5 than for the other dyes may be attributed to its simpler molecular structure and lower molecular weight, in contrast to the more complex functional groups found in the SNB, BRF‐X, and STGB dyes. Further experiments were conducted with the RB5 dye, as its removal percentage was greater than that of the other three dyes.

### 3.4 Effect of pH on Dye Biosorption

The optimum pH for biosorption was determined by testing seven different pH values ranging from 3.0 to 9.0. The data were collected after 10 min of incubation from each setup. The results from these trials were summarized in Table 2. The maximum removal percentage was recorded as 95.0% at pH 3.0, which is the acidic end of the pH scale tested in this study. This result demonstrates the pivotal role of hydrogen ions in effective biosorption in our setup. As a result, further experiments were conducted with the media at pH 3.0. On the other hand, approximately 6% removal still occurs at basic pH levels despite the electrostatic repulsive forces among the dye molecules, biosorbent extracellular surface, and free hydroxyl groups in the environment. This observation can be evaluated because there may be more than one removal mechanism.

**TABLE 2:** Effect of pH on RB5 biosorption (pH: 3–9; *C*_0_: 23.3 mg/L; *T*: 30°C; volume: 40 mL; biomass dose: 0.25 g/L).

pH	Removal %
3	95.0 ± 0.4
4	30.7 ± 0.5
5	12.0 ± 0.9
6	9.30 ± 0.9
7	6.90 ± 1.2
8	6.10 ± 0.6
9	6.40 ± 0.5

pH is a critical parameter for biosorption capacity. Changes in pH affect the binding of dye molecules to adsorbents, as the surface charge of the adsorbent is directly influenced by pH (Kamalian et al. 2024). Sulejmanović et al. (2023) investigated the removal capacity of Eriochrome Black T (EBT) dye using a biosorbent derived from pomegranate peel. They reported a maximum adsorption capacity of 15.50 mg/g at pH 3. This effect was attributed to the electrostatic attraction between the positively charged adsorbent and the negatively charged EBT. The reduction in removal efficiency when the pH increased from 3 to 6 was explained by the decreased proportion of positively charged functional groups on the adsorbent surface.

**FIGURE 5:**
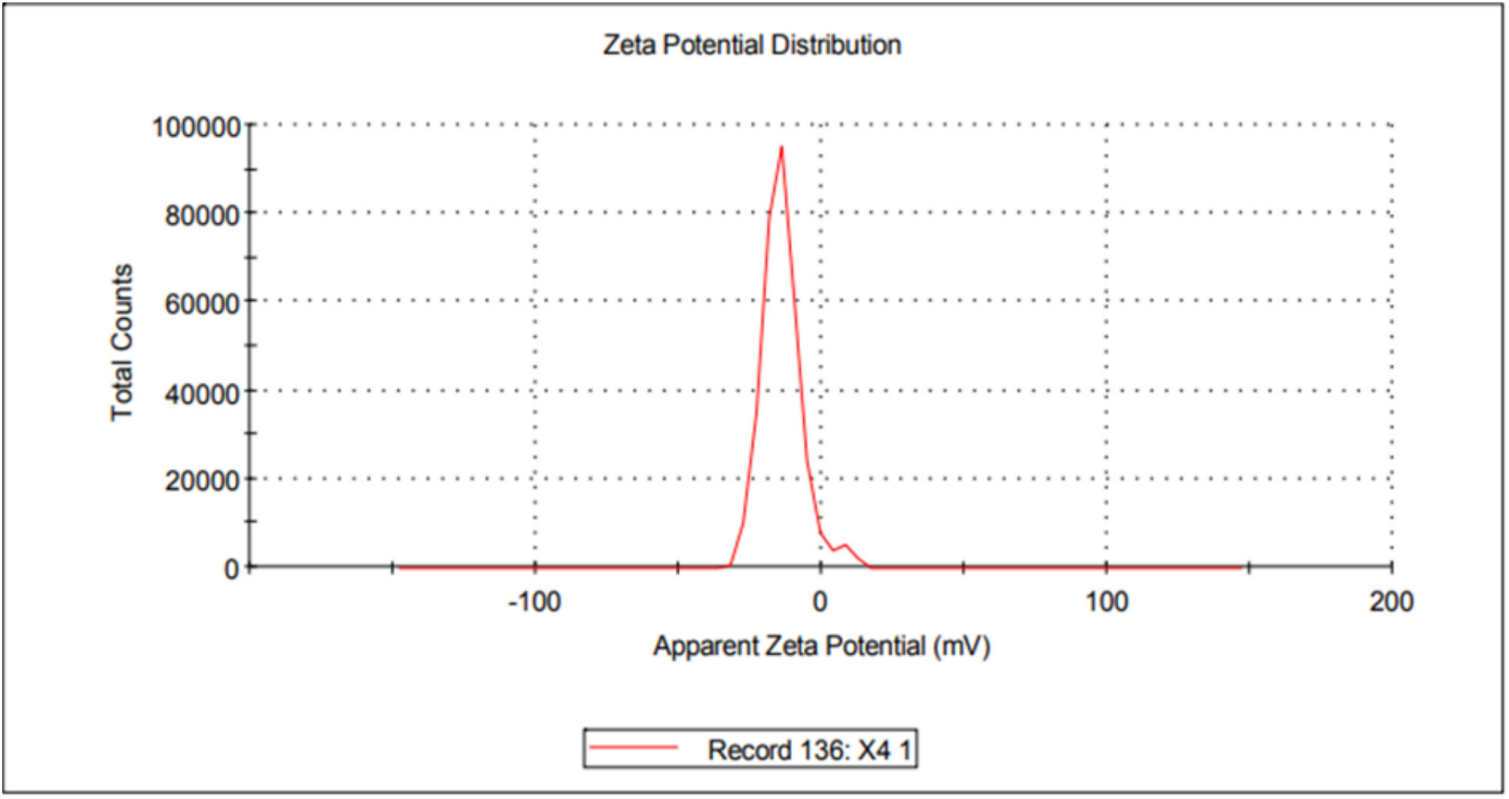
Zeta potential distribution of dry *L. adecarboxylata* biomass.

The Reactive Black 5 dye contains sulfonic (SO_3_^−^) groups and has a negative charge in aqueous media (Tonato et al. 2019; Mao et al. 2009). At low pH values, the bacterial surface is positively charged (Rizvi et al. 2020), which facilitates electrostatic attraction between the biomass and the dye (Mao et al. 2009). This situation may account for the occurrence of maximum biosorption within strongly acidic pH ranges. Under acidic conditions, excess H^+^ ions protonate functional groups on the biosorbent, imparting a positive charge that promotes electrostatic attraction with anionic dye molecules. As the pH increased, the declining H^+^ ion concentration adversely affected the adsorption capacity. The biosorbent zeta potential was measured −12.6 ± 1.1 mV (Figure 5). As the pH decreased, the positive charge on the surface increased, enhancing electrostatic attractions and thereby increasing the adsorption capacity (Kamalian et al. 2024; Yenikaya et al. 2010). These findings highlighted the significant role of the zeta potential in biosorption processes in relation to pH.

In another study, Reactive Black 5 (RB5) and Direct Red 23 (DR23) dyes were removed via copper oxide nanoparticles synthesized from *Stenotrophomonas* sp. bacteria. The maximum biosorption capacity for both dyes was achieved at an acidic pH of 2.0. This was attributed to the increased H^+^ concentration under acidic conditions, which reduced the zeta potential of the adsorbent and increased the positive charge on the surface due to the protonation of functional groups, thereby increasing electrostatic attraction between the dye and the adsorbent. Additionally, as the pH increased, the competition for binding sites intensified, leading to a decrease in the adsorption capacity (Kamalian et al. 2024). In this study, a decrease in biosorption capacity was observed with increasing pH.

### 3.5 Effects of Different Biosorbent Doses on Dye Biosorption

**FIGURE 6:**
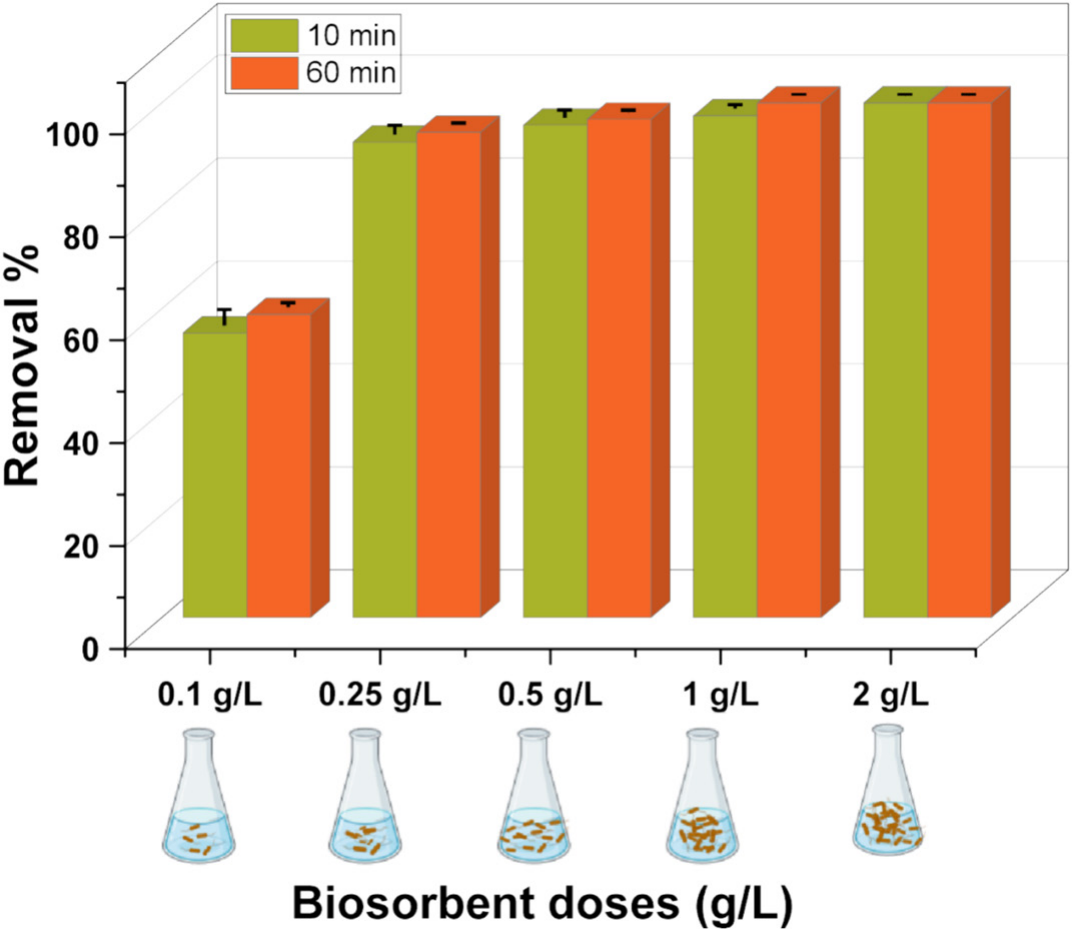
Biosorption by *L. adecarboxylata* biomass in aqueous solutions with various biosorbent doses (pH: 3; *C*_0_: 27.2 mg/L; *T*: 30°C; volume: 40 mL; biomass dose: 0.1–2.0 g/L).

To determine the effect of the biosorbent concentration on the biosorption capacity, experiments were performed in a 27.2 mg/L RB5 solution at pH 3.0 with biosorbent doses ranging from 0.1–2.0 g/L added sequentially. The dye removal process in each medium was measured after 10 min and 60 min, and the results were shown in Figure 6.

As shown in Figure 6, at a biosorbent dose of 0.1 g/L, the removal percentage was 55.3% after 10 min and 58.9% after 60 min. Increasing the biosorbent dosage by 2.5 times (from 0.1 to 0.25 g/L) resulted in a significant increase in the RB5 removal percentage from 55.3% to 92.4% after 10 min. When the biomass dose was increased by five times (0.5 g/L), the removal percentage reached 96.8% at 60 min. At higher biosorbent dosages, only minimal increases in removal percentages were observed. In the present study, although 100% dye removal was achieved in 60 min with a biomass dosage of 1.0 g/L, the time required to reach 100% dye removal was shortened in media with a biomass dosage of 2.0 g/L, and all dye was removed from the medium within 10 min. For this reason, further experiments were performed in the media with a biomass concentration of 2.0 g/L. Our findings are also in line with those of previous studies. For example, Kamalian et al. (2024) investigated the biosorption of RB5 with using copper oxide nanoparticles. In their study, increasing the biosorbent dosage improved the dye removal efficiency; however, after saturation was reached, further removal did not significantly increase. Higher quantities of adsorbent facilitate the interaction of additional active sites, which results in increased adsorption capacity. However, once the optimal dosage level is reached, saturation between the dye molecules and the biosorbent surface occurs, leading to stabilization of the dye removal efficiency (Jeyavishnu and Alagesan 2020). Kalpana et al. (2020) investigated the biosorption capacity of Malachite Green (MG) using *Exiguobacterium* sp. VK1. In their study, the effect of the biosorbent dosage on MG removal was evaluated with respect to biomass concentrations ranging from 0.2 to 2 g/L. Increasing the dosage from 0.2 to 0.5 g/L resulted in a notable increase in the MG removal efficiency from 62.05% to 73.4%. However, further increases in dosage did not significantly enhance the removal efficiency; this was attributed to the increase in diffusion path length, which led to an almost constant removal rate for MG dye molecules.

### 3.6 Effect of Increasing Dye Concentrations on Biosorption

**FIGURE 7:**
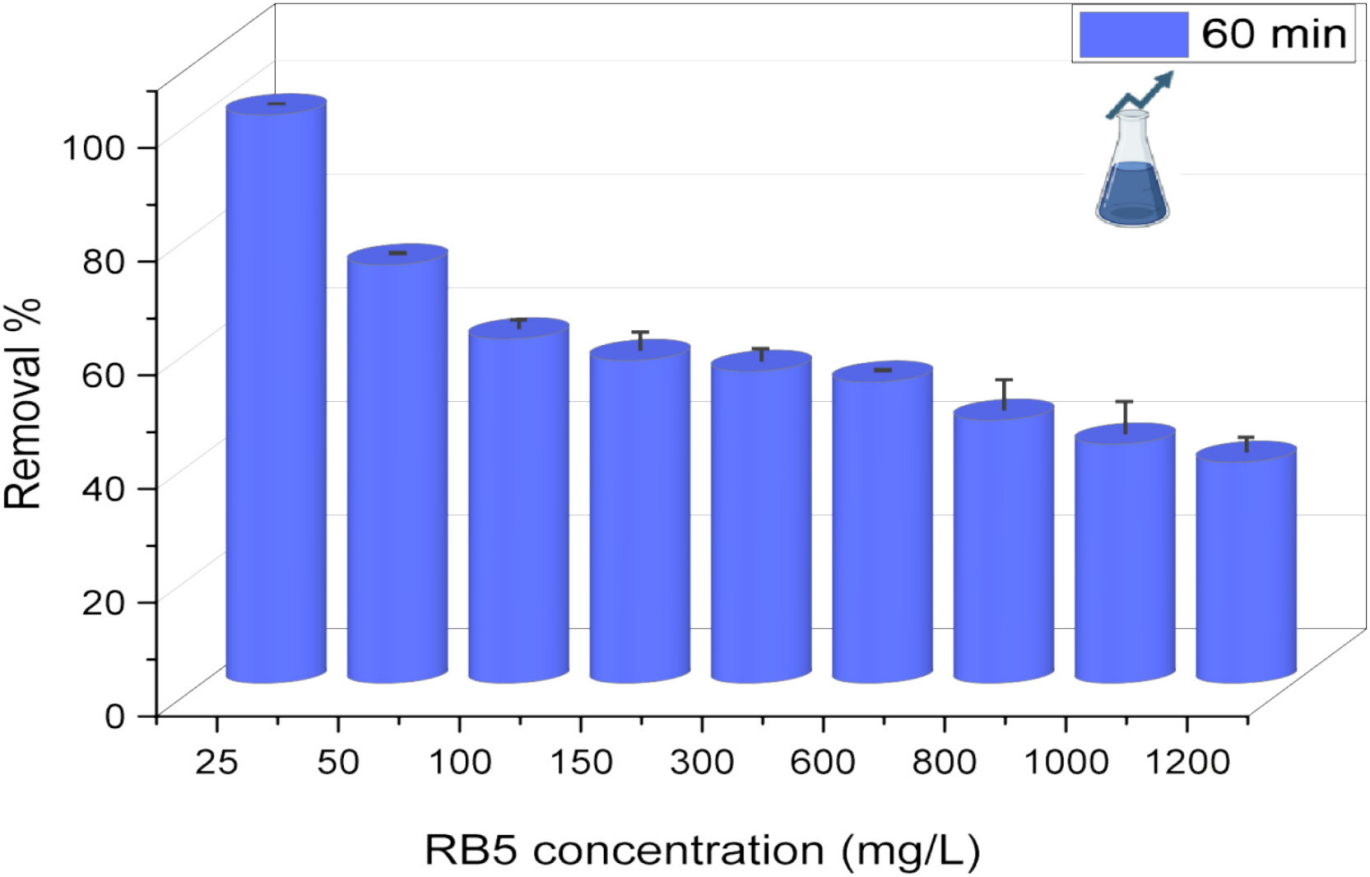
Biosorption of various RB5 concentrations by *L. adecarboxylata* biomass in aqueous solution (pH: 3; *C*_0_: 25–1200 mg/L; *T*: 30°C; volume: 40 mL; biomass dose: 2.0 g/L).

To determine the effect of the RB5 concentration on the biosorption capacity, nine different dye concentrations ranging from 25 to 1200 mg/L were added to media, each containing 2.0 g/L biomass at pH 3.0. The residual dye concentration was measured after 60 min. As shown in Figure 7, at an initial RB5 concentration of 25 mg/L, the removal percentage was 100%. However, increasing the dye concentration subsequently decreased the level of dye removal. For example, when the dye concentration was doubled (50 mg/L), the removal percentage decreased to 73.6%. At a concentration of 100 mg/L RB5, the removal percentage was 60.6%. At a concentration of 150 mg/L, the removal percentage was 56.8%. When the RB5 concentration increased to 300 mg/L, the removal percentage was 54.9%. At concentrations of 600, 800, 1000, and 1200 mg/L RB5, the removal percentages were 53%, 46.3%, 42.1%, and 38.9%, respectively.

With increasing initial RB5 concentration, the dye removal efficiency gradually decreased. These data were supported by previous reports (Muthulakshmi et al. 2021). For example, Kamalian et al. (2024) investigated the removal of RB5 via the use of copper oxide nanoparticles. In their study, increasing the dye concentration from 5 to 80 mg/L reduced the removal percentage from 53% to 9% because of the binding of the contaminant to the active sites of the biosorbent, thus preventing further adsorption of the dye beyond a certain concentration (Unal et al. 2019). This occurs because, at lower concentrations, the ratio of the initial dye concentration to the accessible surface area is relatively low, leading to fractional biosorption that remains unaffected by the initial concentration (Vijayaraghavan et al. 2007). Conversely, at higher concentrations, the number of available biosorption sites becomes limited relative to the quantity of dye molecules present, resulting in the removal of RB5, which depends on the initial dye concentration (Khattri and Singh 1998; Crini and Badot 2008). In our study, removal decreased in a similar manner as the RB5 concentration increased. Nevertheless, the removal percentages recorded in this study are relatively higher than those reported in previous studies, even at high initial dye concentrations. The decrease in the RB5 removal with increasing initial dye concentration is attributed to the saturation of adsorption sites on the biosorbent surface, leading to the active binding regions on the biosorbent being insufficient in comparison to the increasing number of dye molecules.

A comparison of the RB5 adsorption capacity of different sorbents and their optimum parameters was shown in Table 3. When this study was compared with previous studies, the highest removal (100%) was achieved in the shortest contact time (10 min) with the current study.

**TABLE 3:** Comparison of different sorbents for the biosorption of RB5 with the findings of this study.

Sorbent	Dye	Adsorption capacity (%)	Optimal parameters	References
Biogenic copper oxide nanoparticles	RB5	94%	pH: 2.0 Sorbent dose: 0.08 g Contact time: 80 min Dye concentration: 20 mg/L	Kamalian et al. (2024 )
Cetyl trimethyl ammonium bromide modified *Aspergillus versicolor* biomass	RB5	98%	pH: 2.0 Sorbent dose: 5 mg/L Contact time: 420 min Dye concentration: 200 mg/L	Huang et al. (2016 )
Peanut shells	RB5	95.3%	pH: 2.0 Sorbent dose: 5 g Contact time: 15 min Dye concentration: 10 mg/L	Çelebi et al. (2024 )
Potato peel waste	RB5	85.5%	pH: 3.0 Sorbent dose: 1 g Contact time: 60 min Dye concentration: 50 mg/L	Samarghandy et al. (2011 )
*L. adecarboxylata* dried biomass	RB5	100%	pH: 3.0 Sorbent dose: 2 g/L Contact time: 10 min Dye concentration: 25 mg/L	Present study

### 3.7 Fourier Transform Infrared Spectroscopy (FTIR) Analysis

**FIGURE 8:**
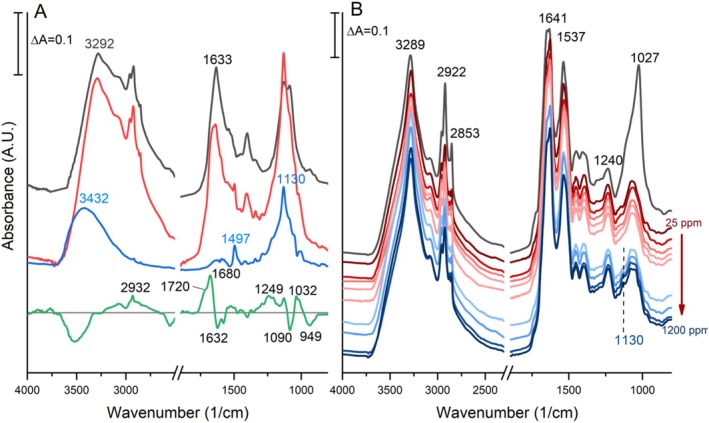
(A) Infrared spectra of the biomass supernatant before adsorption (gray) and after adsorption (red), overlaid with the spectrum of the dye (blue). The difference spectrum (green) was calculated by subtracting the supernatant spectrum before adsorption from that after adsorption to display the active functional groups. (B) Overlaid spectra of biomass pellets before adsorption (gray) and after adsorption (shades of red and blue). All the spectra in both panels are shifted arbitrarily along the vertical axis for better visual comparison.

The FTIR spectra of the pellet and the supernatant obtained from the biomass were analyzed separately before and after the adsorption process to identify the functional groups involved in adsorption. The contribution of dye molecules to the biomass spectrum had to be differentiated by first identifying the peaks of the dye (Figure 8A, blue trace). The spectrum of the dye was characterized mainly by a peak at 1130 cm^−1^ assigned to sulfate groups, which are highly repeating units in the structure. The relatively wide peak at 3432 cm^−1^ was assigned to the N–H and O–H stretching modes. The small but sharp peak at 1497 cm^−1^ was assigned to the C–N stretching and N–H bending modes.

During centrifugation, extracellular polymeric substances (EPSs) detach from the cell surface and can be harvested from the supernatant (Li et al. 2021). Thus, the infrared spectrum of the supernatant was used to study the functional groups of EPS. The structure of EPS expressed by *L. adecarboxylata* shows functional groups in the range of 3500–3000 cm^−1^, with a peak at ~3290 cm^−1^ corresponding mainly to the N–H and O–H stretching modes. Lipid chain modes are observed in the range of 3000–2800 cm^−1^. In the low‐frequency region, the regions 1700–1600, 1600–1500, 1500–1300, and 1200–1000 cm^−1^ were assigned to protein Amide I, Amide II, carboxyl groups and hydrocarbons, and polysaccharides and nucleic acids, respectively (Shi et al. 2023; Cakmak‐Arslan et al. 2024). These assignments suggest that the soluble EPS of *L. adecarboxylata* was composed of proteins, lipids, nucleic acids, and polysaccharides. Figure 8A (gray vs. red traces) showed that the spectrum of EPS changes with dye treatment. The elevated absorbances at 3292, 1500, and 1130 cm^−1^ suggest that the dye molecules interact with the EPS structures.

A difference spectrum was calculated to observe the functional groups that take part in the adsorption process. The spectrum of EPS before adsorption and the spectrum of dye were subtracted from the EPS spectrum after adsorption. Thus, the positive features in the difference spectrum represent new vibrational modes due to the adsorption. Conversely, the negative features represent diminished or declined modes. The positive peak at 2932 cm^−1^ indicates the involvement of lipid acyl chain groups in adsorption. Another lipid‐originating positive peak at 1720 cm^−1^ corresponds to the protonation of ester carboxyl groups. The positive 1680 cm^−1^ and negative 1580 cm^−1^ peaks represent the C=O and COO^−^ modes, respectively. Both peaks indicate the protonation of other carboxyl groups, such as those in aspartic and glutamic acid. The region in the range 1500–1200 cm^−1^ shows various C–H modes. This positive region might indicate interactions between the partners via H‐bonding, thus leading to the formation of hydrocarbons. The negative 1090 cm^−1^ and positive 1032 cm^−1^ peaks suggest the protonation of sulfate groups in the dye molecules as a result of the adsorption. A comparative analysis of the pellet samples before and after adsorption revealed no visible difference in the dye concentration from 25 to 300 ppm (Figure 8B). For the biomass treated with 600, 800, 1000, and 1200 ppm, a gradual increase in the peak at 1130 cm^−1^ was observed. For these higher dye concentrations, the spectra of pellets show evidence that some of the dye molecules attach to the bacterial surface, possibly to more tightly bound EPS structures that were not separated by centrifugation (Li et al. 2021). However, the functional groups on the bacterial surface involved in these interactions could not be detected. The spectral signatures of these functional groups are either too small to be detected or obscured by other larger peaks.

### 3.8 Adsorption Kinetic Studies

**FIGURE 9:**
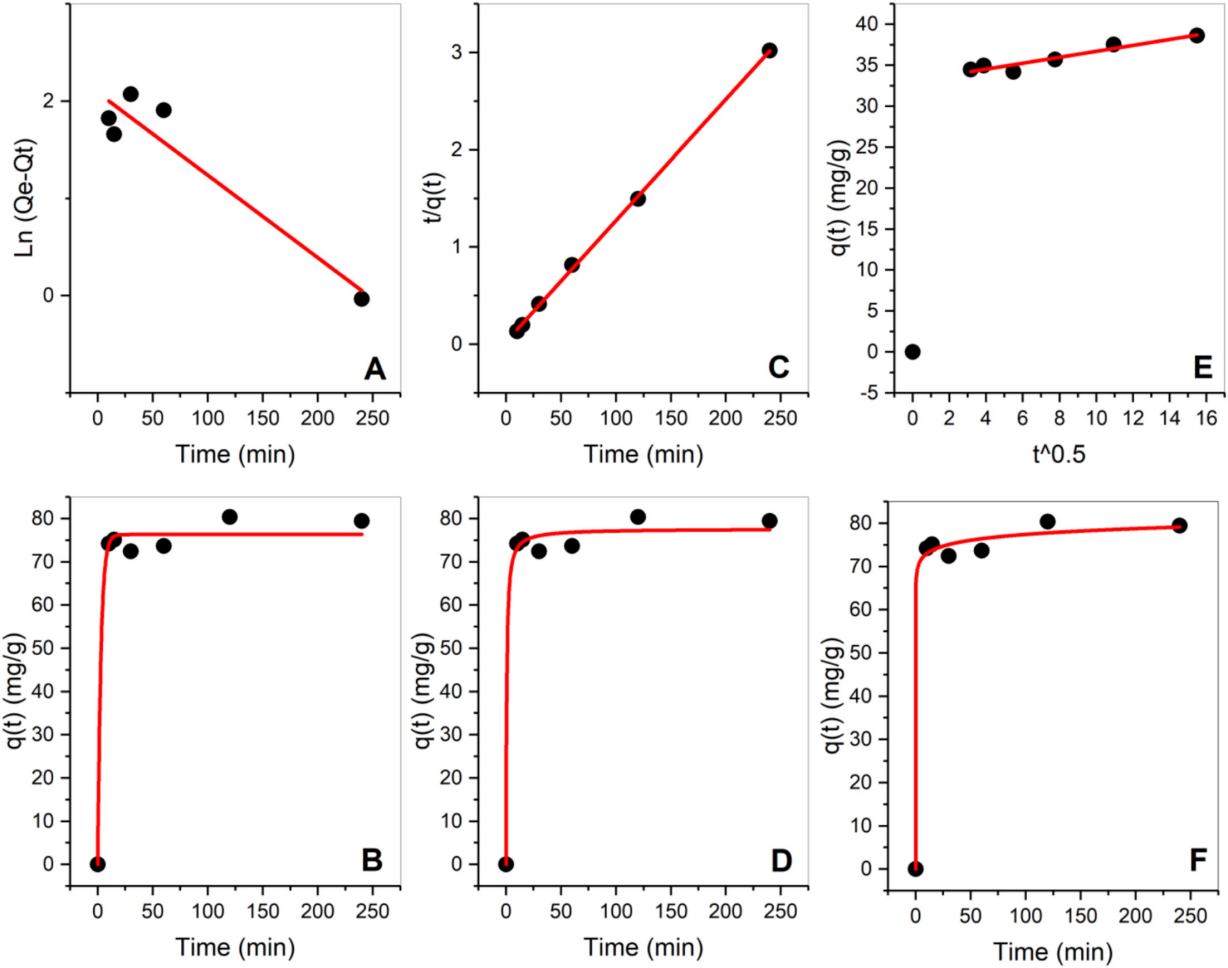
Adsorption kinetics of Black B on *L. adecarboxylata* . (A) Linear pseudo‐first‐order, (B) nonlinear pseudo‐first‐order, (C) linear pseudo‐second order, (D) nonlinear pseudo‐second‐order, (E) linear intraparticle diffusion (IPD), and (F) Elovich models.

The adsorption kinetics fitting models and their respective parameters, depicted in Figure 9 and Table 4, were utilized to assess the kinetics of RB5 adsorption. In this study, both linear and nonlinear models were employed. In the past two decades, most studies have employed the classical PFO and PSO rate laws to model kinetic data across a diverse range of adsorption systems, including those involving biomass, nanomaterials, heavy metals, and pharmaceuticals (Revellame et al. 2020). Adsorption kinetics, crucial for defining adsorption performance, describe solute uptake over time. Compared with the models' coefficient of determination (*R* ^2^), reduced chi‐square ($\chi_{\mathrm{red}}^{2}$), and residual sum of squares (RSS) scores, the PSO kinetic model better fits the experimental data, as evidenced by the calculated *q*_*e*_ values. Specifically, the PSO linear model yielded a *q*_*e*_ of 80.0 mg/g, and the nonlinear model yielded 77.64 mg/g, which was closer to the experimental *q*_*e*_ of 80.4 mg/g, whereas the PFO model's *q*_*e*_ values were 76.33 and 124.02 mg/g for the nonlinear and linear models, respectively. The pseudo‐second‐order kinetic model assumes that the rate is second‐order and that the rate‐limiting step is chemical adsorption (Pathirana et al. 2020). This alignment underscores the enhanced explanatory power of the PSO model in describing RB5 dye adsorption kinetics over time. Recent studies suggest that nonlinear models, including PSO, often provide superior fits. Huang et al. (2016) employed cetyl trimethylammonium bromide (CTAB)‐modified *Aspergillus versicolor* biomass (AVB) as a biosorbent for the biosorption of RB5 dye. In this study, the pseudo‐second‐order kinetic model was preferred for the biosorption of RB5 onto the modified AVB. In another study, the adsorption kinetics of RB5 dye using biogenic copper oxide nanoparticles synthesized from *Stenotrophomonas* sp. were examined, and it was found that the pseudo‐second‐order model better described the adsorption process (Kamalian et al. 2024).

**TABLE 4:** Kinetic analysis of RB5 adsorption.

Experimental	*q*_*e*_	80.4 (mg/g)
Nonlinear PFO	*q*_*e*_	76.33 ± 1.47 (mg/g)
	*K*_1_	0.347 ± 0.145
	*R* ^2^	0.9902
	$\chi_{\mathrm{red}}^{2}$	9.759
	RSS	48.795
Linear PFO	*q*_*e*_	124.02 (mg/g)
	*K*_1_	0.0196
	*R* ^2^	0.9012
	$\chi_{\mathrm{red}}^{2}$	0.0979
	RSS	0.294
Nonlinear PSO	*q*_*e*_	77.64 ± 1.724 (mg/g)
	*K*_2_	0.0206 ± 0.0157
	*R* ^2^	0.9923
	$\chi_{\mathrm{red}}^{2}$	7.69
	RSS	38.45
Linear PSO	*q*_*e*_	80.0 (mg/g)
	*K*_2_	0.0059
	*R* ^2^	0.9995
	$\chi_{\mathrm{red}}^{2}$	0.009
	RSS	0.0031
IPD	*K*_*Diff*_	0.36235
	*C*	33.1
	*R* ^2^	0.9164
	$\chi_{\mathrm{red}}^{2}$	128.31
	RSS	1.336
Elovich	*α*	(4.21 ± 0.7) × 10^15^
	*β*	0.52 ± 0.22
	*R* ^2^	0.9950
	$\chi_{\mathrm{red}}^{2}$	4.96
	RSS	24.82

Previous studies, such as that of Muthulakshmi et al. (2021), similarly advocated for the suitability of the PSO model in kinetic studies. The PSO model posits that adsorption occurs via chemical mechanisms involving varying binding energies across active sites and often outperforms in predicting biological adsorption kinetics (Shi et al. 2023). The Elovich model is often used to describe chemisorption processes on highly heterogeneous surfaces (Pan et al. 2017). The high conformity to the Elovich and pseudo‐second‐order kinetic models indicates that chemical interactions dominate the adsorption mechanism (Song et al. 2023). A good fit indicates that the biosorption process may involve chemical interactions between the dye molecules and the bacterial biomass. The IPD model shows two linear patterns, suggesting two‐step adsorption kinetics (Jathanna et al. 2023). The initial section with a high slope represents the bulk or surface diffusion that occurs very rapidly. The next phase is slower owing to the smaller slope. It represents the pore diffusion or intraparticle diffusion‐controlled zone. However, the low *R* ^2^ score in this model suggests that the adsorption involves multiple processes and is not dominated by the intraparticle diffusion (Wang and Guo 2022).

### 3.9 Interpretation of Isotherm Models for the Biosorption of Black B

**FIGURE 10:**
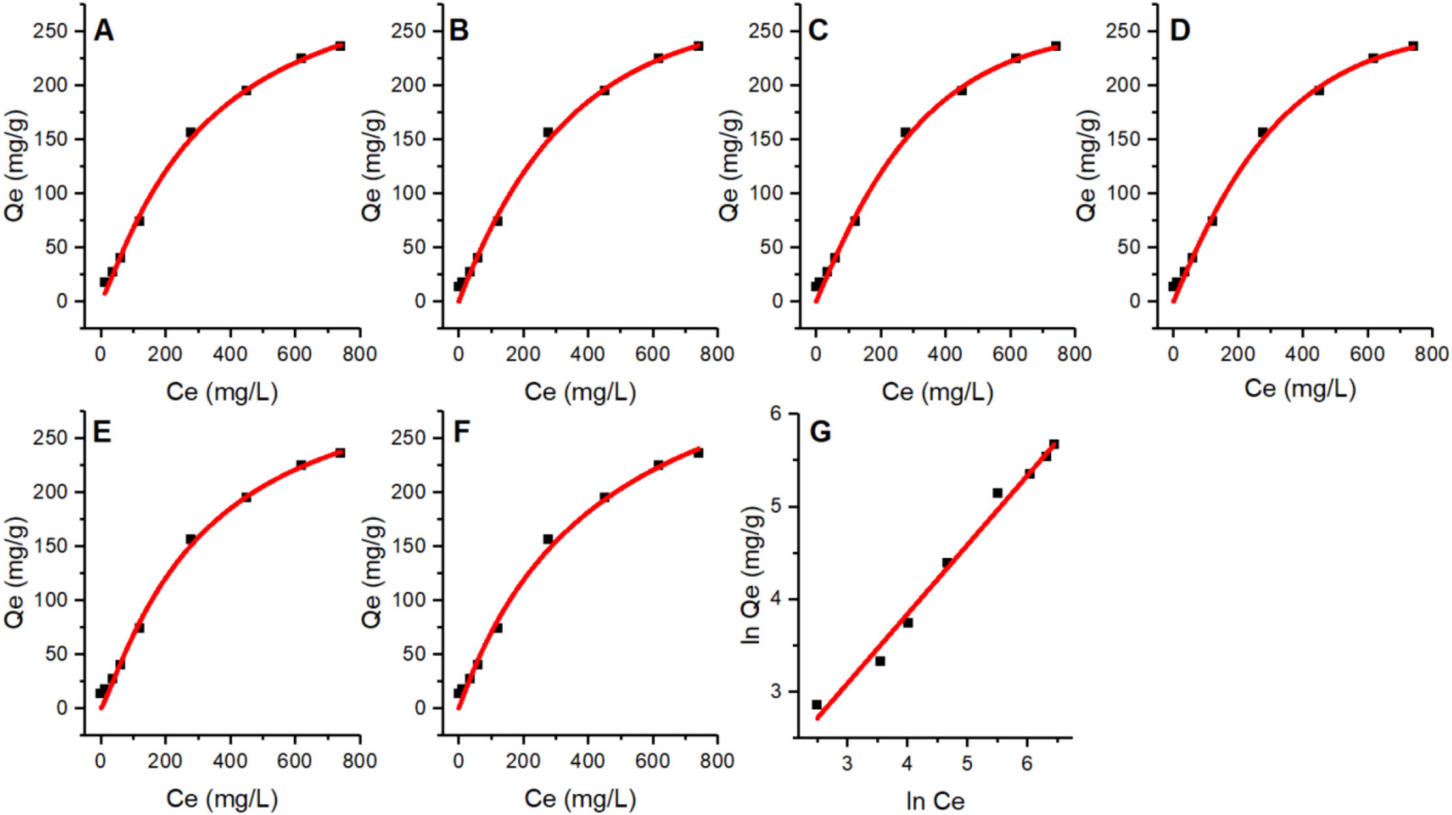
Isotherm curve fitting of the adsorption of the RB5 dye by *L. adecarboxylata* via the Hill (A), Jovanovich (B), Toth (C), Redlich–Peterson (D), Sips (E), Langmuir (F), nonlinear equations, and the Halsey (G) linear equation.

Various isotherm models were applied to fit the experimental data obtained from the removal of dye molecules by the bacterial biomass (Table 5 and Figure 10). The Hill isotherm model yielded the best fit, with an *n* value of 1.56, followed by the Jovanovich, Toth, Redlich–Peterson, Sips, Langmuir, and Elovich models. The Freundlich, Halsey, and Temkin models did not fit very well. The Hill isotherm model, with an *n* value of 1.56, indicates a cooperative binding process (Torkia et al. 2014). This suggests that the binding of dye molecules to the bacterial biomass is not independent; rather, the binding of one molecule affects the binding of other molecules. An *n* value greater than one implies positive cooperativity, meaning that the binding of one dye molecule enhances the likelihood of subsequent molecule binding. This model's excellent fit suggests that the bacterial biomass has multiple binding sites that interact cooperatively, leading to efficient dye removal.

**TABLE 5:** Results of the isotherm curve fitting of the adsorption of RB5 by *L. adecarboxylata* .

Hill		Jovanovic		Toth		Redlich–Peterson	
*q*_*m*_	278.40 ± 11.82	*q*_*m*_	266.75 ± 12.78	*q*_*m*_	634.37 ± 185.98	*K*_*R*_	0.714 ± 0.093
*K*_*H*_	5944.3 ± 90.57	*K*_*J*_	(29.6 ± 3.18)10^−4^	*K*_*T*_	(15.8 ± 3.0) 10^−4^	*a*	(1.15 ± 3.06)10^−4^
*n*_*H*_	1.56 ± 0.12			*t*	1.40	*n*_*R*_	1.41 ± 0.37
*R* ^2^ (COD)	0.9993	*R* ^2^ (COD)	0.9948	*R* ^2^ (COD)	0.9951	*R* ^2^ (COD)	0.9951
$\chi_{\mathrm{red}}^{2}$	9.84	$\chi_{\mathrm{red}}^{2}$	51.45	$\chi_{\mathrm{red}}^{2}$	56.80	$\chi_{\mathrm{red}}^{2}$	56.80
HYBRID	1230	HYBRID	18,007	HYBRID	11,359	HYBRID	11,359

Sips		Langmuir		Temkin		Halsey (linear)	
*q*_*m*_	326.95 ± 47.77	*q*_*m*_	386.14 ± 30.62	*A*_*T*_	0.053 ± 0.014	*K*_*Ha*_	0.747 ± 0.034
*K*_*S*_	(31.5 ± 9.5) 10^−4^	*K*_*L*_	(22.2 ± 3.65) 10^−4^	*b*_*T*_	41.61 ± 6.80	*n*_*HA*_	0.343
*n*	1.16 ± 0.16						
*R* ^2^ (COD)	0.9944	*R* ^2^ (COD)	0.9936	*R* ^2^ (COD)	0.9286	*R* ^2^ (COD)	0.9876
$\chi_{\mathrm{red}}^{2}$	64.27	$\chi_{\mathrm{red}}^{2}$	63.54	$\chi_{\mathrm{red}}^{2}$	707.17	$\chi_{\mathrm{red}}^{2}$	
HYBRID	12,854	HYBRID	22,238	HYBRID	247,508	HYBRID	5.05

Freundlich	
*K*_*F*_	4.00 ± 1.4
*n*	1.60 ± 0.14
*R* ^2^ (COD)	0.9815
$\chi_{\mathrm{red}}^{2}$	182.85
HYBRID	63,996

The next best fits were provided by the Jovanovic, Toth, Redlich–Peterson, Sips, Langmuir, and Elovich models. Each of these models offers unique insights into the biosorption process. The Jovanovic and Langmuir models assume monolayer adsorption onto a surface with a finite number of identical sites. The Jovanovic model differs from the Langmuir model in that it excludes lateral interactions (Hadi et al. 2010). Both models yield similar equilibrium constants. On the other hand, the maximum adsorbed concentration (*q*_*m*_) values differ significantly. When the separation factor (*R*_*L*_) is calculated to examine the favourability of the adsorption process, the result lies between 0 and 1, indicating a favorable adsorption process (Al‐Ghouti and Da'ana 2020). A reasonable fit with these two models suggests that the bacterial biomass has a fair number of uniform binding sites with favorable adsorption. The Toth model is an empirical isotherm that accounts for heterogeneity in biosorption sites. The *t* value is different from unity, confirming the heterogeneity of the adsorption mechanism (Podder and Majumder 2016). A good fit with this model suggests that the bacterial biomass has a heterogeneous surface with varying affinities for the dye molecules. The Redlich–Peterson model is a hybrid model that combines the Langmuir and Freundlich isotherms, indicating that the biosorption process may involve both monolayer adsorption and heterogeneous surface energies. Like the Redlich–Peterson model, the Sips model combines Langmuir and Freundlich characteristics, but it is particularly useful for describing adsorption on heterogeneous surfaces at high concentrations.

The most prominent strength of the present study lies in the achievement of 100% removal in very a short time. Additionally, the high biomass production capacity of the *L. adecarboxylata* played a crucial role in enhancing the overall biosorption process. This strain demonstrated approximately 50% removal even at all the tested RB5 concentrations (600, 800, 1000, and 1200 mg/L), highlighting its significant potential for biosorption applications. However, there may also be conditions that limited the removal potential of the tested biosorbent. For example, given the variability of pH values in real wastewater, depending on their sources, pH adjustment is frequently necessary, constituting an additional step that could increase treatment costs. Nonetheless, for wastewaters naturally exhibiting low pH values, the experimental conditions utilized in this study may provide a more cost‐effective treatment strategy, obviating the need for prior pH adjustment.

The most prominent strength of the present study lies in the achievement of 100% removal very a short time. Additionally, the high biomass production capacity of the *L. adecarboxylata* played a crucial role in enhancing the overall biosorption process. This strain demonstrated approximately 50% removal even at all the tested RB5 concentrations (600, 800, 1000, and 1200 mg/L), highlighting its significant potential for biosorption applications. However, there may also be conditions that limited the removal potential of the tested biosorbent. For example, given the variability of pH values in real wastewater, depending on their sources, pH adjustment is frequently necessary, constituting an additional step that could increase treatment costs. Nonetheless, for wastewaters naturally exhibiting low pH values, the experimental conditions utilized in this study may provide a more cost‐effective treatment strategy, obviating the need for prior pH adjustment.

## 4 Conclusion

Industrial wastewater should not be discharged without proper treatment. In addition to many traditional treatment techniques, biological treatment based on bioremediation is necessary. Among these techniques, biosorption is a simple, cost‐effective, and environmentally friendly method. In our study, the X3 strain isolated from the Düden Waterfall (Antalya, Türkiye) was identified by 16S rDNA analysis as *L. adecarboxylata* (GenBank no: PP886272). The biosorbent of *L. adecarboxylata* was evaluated for the removal of four reactive dyes, and it exhibited the highest efficiency in removing the RB5 dye, achieving 96.5% removal in 10 min. During trials with seven different pH values (3–9), the highest biosorption was achieved at pH 3.0, with 95% removal. When the effect of five different biomass concentrations (0.1–2.0 g/L) on biosorption was investigated, by increasing the biomass 2.5‐fold (from 0.1 to 0.25 g/L), biosorption increased from 55.3% to 92.4% after 10‐min contact time. However, at 2.0 g/L biomass concentration, 100% removal was reached at the 10th minute. When the effect of initial RB5 concentration on biosorption was examined, 100% removal was observed at 25 mg/L RB5 concentration, whereas 73.6% removal was achieved at 50 mg/L after 10 min. In conclusion, the complete biosorption (100%) was found in aqueous solution with 2.0 g/L biosorbent, 27.2 mg/L RB5, at pH 3.0 after 10 min. The same removal efficiency was also observed with half of the biomass, but the process required a longer time. FTIR analysis of the soluble extracellular polymeric substances (EPS) from *L. adecarboxylata* revealed that the EPS plays the main role in removing the dye and is composed of proteins, lipids, nucleic acids, and polysaccharides. The sulfonate groups in the dye structure form H‐bonds with the negatively charged groups on the EPS. Although the biomass pellet does not seem to play a role in biosorption at low concentrations of dye (< 600 ppm), higher concentrations were observed to attach to the surface. The zeta potential (−12.6 ± 1.1 mV) revealed electrical properties and charge distributions on the biosorbent surface, highlighting its crucial role in the biosorption process. Kinetic analysis revealed that the adsorption of RB5 followed a pseudo‐second‐order kinetic model, suggesting a chemical interaction between RB5 and the biomass. This result is consistent with the findings of the FTIR analysis. The excellent fit of the Hill isotherm model, along with the reasonable fits of the Jovanovich, Toth, Redlich–Peterson, Sips, Langmuir, and Elovich models, suggests that the biosorption of RB5 by bacterial biomass involves a complex interplay of cooperative binding, heterogeneous surface interactions, and possibly chemisorption. The poor fit of the Freundlich, Halsey, and Temkin models further supports the notion that the biosorption process is not purely heterogeneous or multilayered. Overall, functional groups characterization and the nature of interactions revealed by FTIR spectroscopy agree well with the adsorption mechanism suggested by the isotherm models. In addition to the success in the curve fitting, consistency with other experimental and analytical results is also important for the correct identification of the adsorption mechanism (Scheufele et al. 2020). These insights can guide future studies and optimization of biosorption processes for efficient RB5 removal. This study revealed that dry *L. adecarboxylata* biomass is a cost‐effective biological removal agent that provides significant advantages in terms of environmental sustainability and economic efficiency. In addition, it is a powerful biosorbent candidate that can be used to treat real wastewater containing dyes.

In our study, *L. adecarboxylata* biosorbent, which showed high biosorption capacity, can be used in future research. For example, *L. adecarboxylata* biomass, which effectively removes RB5, can be used to remove different pollutants. Moreover, changing the surface charges of this effective biosorbent and taking advantage of its unique surface morphology can facilitate the effective removal of almost all pollutants. However, the tested biosorbent may also find potential application in real wastewater.

## Author Contributions

**Seda Şen:** formal analysis, investigation, project administration, software, writing – original draft. **Filiz Korkmaz:** data curation, formal analysis, investigation, software, writing – original draft, writing – review and editing. **Nur Koçberber Kiliç:** data curation, investigation, project administration, supervision, validation, writing – review and editing.

## Conflicts of Interest

The authors declare no conflicts of interest.

## Data Availability

The data that support the findings of this study are available from the corresponding author upon reasonable request.
